# Plant Feed Additives as Natural Alternatives to the Use of Synthetic Antioxidant Vitamins in Livestock Animal Products Yield, Quality, and Oxidative Status: A Review

**DOI:** 10.3390/antiox10050780

**Published:** 2021-05-14

**Authors:** Eleni Tsiplakou, Rosario Pitino, Carmen L. Manuelian, Marica Simoni, Christina Mitsiopoulou, Massimo De Marchi, Federico Righi

**Affiliations:** 1 Laboratory of Nutritional Physiology and Feeding, Department of Animal Science, School of Animal Biosciences, Agricultural University of Athens, Iera Odos 75, GR-11855 Athens, Greece; chr_mitsiopoulou28@hotmail.com; 2 Department of Veterinary Science, University of Parma, via del Taglio 10, 43126 Parma, Italy; rosario.pitino@unipr.it (R.P.); marica.simoni@unipr.it (M.S.); federico.righi@unipr.it (F.R.); 3 Department of Agronomy, Food, Natural Resources, Animals and Environment, University of Padova, Viale dell’ Università 16, 35020 Legnaro, Italy; massimo.demarchi@unipd.it

**Keywords:** organic livestock, plant extracts, vitamins, tocopherols, swine, ruminants

## Abstract

The interest for safe and natural foods of animal origin is currently increasing the use of plant feed additives (PFA) as antioxidants in animal nutrition. However, studies with livestock animals dealing with PFA as antioxidants are scarce. The aim of the present review was to evaluate the antioxidant impact of PFA compared with synthetic vitamins on animal food product yield and quality. For this purpose, peer-reviewed studies published between 2000 and 2020 were collected. Most papers were carried out on ruminants (*n* = 13), but PFA were also tested in swine (*n* = 6) and rabbits (*n* = 2). The inclusion of PFA in the diets of pigs, rabbits, and ruminants improved the products’ quality (including organoleptic characteristics and fatty acids profile), oxidative stability, and shelf life, with some impacts also on their yields. The effects of PFA are diverse but often comparable to those of the synthetic antioxidant vitamin E, suggesting their potential as an alternative to this vitamin within the diet.

## 1. Introduction

Currently, consumers are interested in safe and natural foods of animal origin, and in some cases, they are also willing to pay a premium price for them [1,2]. Moreover, the European Union has banned in-feed antibiotics in animal nutrition to prevent antibiotic-resistant bacteria, which has increased the interest in the use of plant feed additives (PFA) as dietary ingredients in animal nutrition [3,4,5]. Moreover, the possibility of using PFA, such as essential oils, plant extracts, and in particular, plant food industry by-products, is of great interest since it is in harmony with the philosophy underlying environmental sustainability by reducing industry wastes [6,7]. Moreover, organic farms are allowed to use industry wastes to feed their animals [8] This feeding strategy allows to convert by-products into high-value food products (e.g., milk and meat) and contributes to the reduction in the feed to food competition in livestock production [6,7], which leads to important technical implications in the whole food chain.

The PFA (phenolic acids, phenolic di-terpenes, flavonoids, and volatile oils) are considered natural antioxidants, because they are able to donate hydrogens and therefore to interrupt the oxidative chain in tissues. Generally, PFA can trap free radicals and chelate metals. The free radical scavenging activity of PFA depends on the location and number of hydroxyl groups and their capacity to donor hydrogens to metals, which inhibit their pro-oxidant activity [9,10]. The PFA also prevents peroxide formation by modifying the activities of antioxidant enzymes. Moreover, they can interact with specific proteins and modulate their expression and activities [11]

By-products derived from the grape and citrus industry contain a wide range of polyphenols [12,13], such as epicatechin, hesperidin, and quercetin, with a great antioxidant potential [14]. Polyphenols revealed an interesting potential to improve animal health and productivity [15,16] due to their antioxidant activity in animals’ organisms and products. Nevertheless, the impact of feeding PFA on the oxidative status of livestock products has been less investigated [17] compared with poultry products. In swine, PFA were mainly evaluated as alternatives to antimicrobial compounds [18]. In ruminants, PFA (in particular essential oils) have been widely investigated as rumen modifiers rather than antioxidants [19,20]. Although different studies have determined the activity of PFA as potential antioxidants, only a few of them have compared their effects with synthetic vitamins.

Therefore, the aim of the present review was to summarize the results available in the literature regarding the potential of antioxidant capacity of PFA—namely plant extract (PE), essential oils (EO), and by-products of plant origin (BP)—compared with synthetic vitamins on yield, quality, and oxidative status of products from livestock animals.

## 2. Materials and Methods

A systematic review of peer-reviewed studies published in Pubmed (www.ncbi.nlm.nih.gov; last accessed on 10 January 2021), ISI Web of Science (www.webofknowledge.com; last accessed on 10 January 2021), and Sciencedirect (www.sciencedirect.com; last accessed on 10 January 2021) databases from January 2000 to December 2020 was performed. Very few papers were published before 2000. In an initial search (conducted in 2018) only 15 papers were found that were in line with our inclusion criteria. Those papers were published between 1976 and 1999. After 2000, papers published on the topic started to increase considerably and the interest in the topic dramatically rose in the last 10–13 years. Therefore, we decided to start the review including papers since 2000.

Studies were selected based on the following criteria: (i) articles comparing the effect of PE or EO with a specific dose of synthetic vitamin or synthetic antioxidants in livestock animals, namely pigs, rabbits, and ruminants; (ii) articles comparing the effect of BP from different agro-industries with a specific dose of synthetic vitamin or synthetic antioxidants in the mentioned species; (iii) articles published in peer-reviewed journals; and (iv) exclude studies dealing with propolis, algae, and additives of animal origin. The keywords used for the search were: plant extract, plant by-product, natural vitamins, synthetic vitamins, swine, livestock, cattle, vitamin E, vitamin C, tocopherols, tocopheryl, antioxidants, organic farming, and organic feeding. A total of 21 papers were retained for the analysis, most of them being conducted on ruminants (*n* = 13), and fewer on swine (*n* = 6) and rabbits (*n* = 2). The majority dealt with essential oils and extracts of rosemary and oregano (*n* = 7), and grape industry by-products (*n* = 5).

## 3. Potential Plant Extracts and Plant By-Products as Alternative Sources of Vitamins for Animal Feeding: Impact on Productivity

### 3.1. Monogastrics

Detailed information for the studies presented in this section is reported in Table S1 in Supplementary Materials.

#### Slaughter and Meat Traits 

Only few studies exist on PFA effects on slaughter and meat traits in monogastrics. The dietary inclusion of vitamin E (VitE, 200 mg/kg) and oregano essential oil (OEO; 0.025% of the diet) for 28 days before slaughter decreased the pH measured 45 min post-mortem (by 3.6% and 4.0%, respectively) of the *Longissimus thoracis* et *lumborum* muscle in pigs exposed to a 5 h transport stress without reaching the pH (6.63) of the unsupplemented and unstressed animals. These differences in the meat pH were not observed at 24 h post-mortem [21]. Moreover, the stress during transportation increased drip loss 24 h post-mortem (by 49.6%) compared with the control group. However, the OEO, and not the VitE, was able to counteract the increase in drip loss related to the transportation. Other meat quality parameters, such as meat color, electrical conductivity, and intramuscular fat, were not affected by transport stress or dietary supplementation [21] (Table 1).

The dietary addition of OEO and Quercetin (QU) during a 28 days trial in finishing pigs also affected some meat traits after 5 h of transportation [22]. In more detail, cold carcass weight and dressing out were higher in OEO than in unsupplemented (8.3% and 8.2%, respectively) and VitE (7.1% and 6.9%, respectively) fed animals [22]. The pH (45 min post-mortem) increased in OEO (by 5.6%) and QU (by 4.9%) groups only compared to the control group, whereas pH (24 h post-mortem) was greater only in QU (by 3.2%) fed pigs. The dietary inclusion of OEO and QU improved meat color (measured by Opto-star color tester) at 24 h post-mortem, in both PFA groups (by 16.9% and 10.8%, respectively), compared with the negative control. Drip loss (at 24 h post-mortem) was also reduced in OEO (by 35.3%) and QU (by 35.9%) compared with the control group. Furthermore, OEO and QU were as effective as VitE in ameliorating the antioxidant status in the pigs’ muscles. The dietary supplementation with OEO and QU, compared with the basal diet, reduced reactive oxygen species (ROS; by 18.0% and 21.0%, respectively) and thiobarbituric acid reactive substances (TBARS; by 19.4% and 20.4%, respectively; Table 1) [22]. Those results suggest that the dietary supplementation with OEO is more effective than VitE in mitigating the consequences of transport stress in finishing pigs.

No significant differences in lipid oxidation (malondialdehyde, MDA) of cooked and raw pig meat were observed when different antioxidant additives (40 mg/kg of rosemary extract (RE), 40 mg/kg of RE plus 2 mg/kg of gallic acid (REG), 40 mg/kg of RE plus 40 mg/kg of VitE (REE), compared with VitE alone (40 mg/kg), were included in a linseed oil-rich diet [23]. Only in raw meat, TBARS values were higher in REG (by 67.6%) than in the negative control group, and in RE (by 129.3%) and REG (by 190.2%) than in the VitE group. No significant effects were reported on some slaughter traits and on the fatty acid composition of the *Longissimus thoracis* muscle of pigs [17].

In another study, pigs were allocated into six different dietary treatments during the weaning (21 days post-weaning until 35 kg of live weight) and finishing (up to 105 kg of live weight) periods [24]. More specifically, the control group was fed a high energy diet without grass meal (GM) in both periods, while the other groups were fed as follow: (i) GM105: pigs fed low-energy diets with GM (100 g GM/kg—weaner, 200 g GM/kg—finisher) from weaning to slaughter; (ii) GM50: pigs fed low-energy diets with GM up to 50 kg of live weight and then a high energy diet from 50 kg to slaughter; (iii) GM80: pigs fed concentrate diet with GM up to 80 kg of live weight and a high nutrient dense finisher diet from 80 kg to slaughter; (iv) VitE: pigs fed concentrate diets with GM up to 80 kg of live weight and then a VitE enriched diet (200 mg/kg) with rapeseed oil (50 g/kg) from 80 kg to slaughter; (v) GTC (green tea catechins), pigs fed concentrate diets with GM up to 80 kg of live weight and then a GTC enriched diet (200 mg tea extracts/kg) with rapeseed oil (50 g/kg) from 80 kg to slaughter. Significant effects were reported on some slaughter traits. The kill-out (carcass weight/slaughter weight) was lower in GTC group than the control (by 1.0%), but higher compared to the GM105 group (by 1.1%), whereas no differences were found compared to the other groups. In addition, the GTC fed animals had higher kill-out (by 10.4%) in comparison with the GM105 ones. The pH 24 h post-mortem of GTC carcasses was lower (by 4.9%) than the GM50 one, whereas fat depth in the shoulder and ham was higher in GTC than GM80 group (by 38.0% and 58.3%, respectively). The chemical composition of the *Longissimus dorsi* (LD) muscle was unaffected by dietary treatments. In GTC-fed animals, a greater ash (by 14.8%) and a lower alpha-tocopherol (by 29.0%) content compared with those consuming GM50 and VitE, respectively, were found. Lipid oxidation of LD muscle was not affected at all under anaerobic conditions. However, lower TBARS values on LD muscle of GTC compared with GM105-fed animals (by 76.9%) were found in modified atmosphere packaging (MAP) after 2 days of storage, and compared with GM105 (by 41.2%) and GM50 (by 44.4%) after 4 days of storage. Nevertheless, neither GTC nor VitE was able to delay lipid oxidation of the LD muscle of pigs after 10 days of storage [24]. The meat fatty acid profile (saturated fatty acids, SFA; monounsaturated fatty acid, MUFA; polyunsaturated fatty acids, PUFA) of LD meat was not modified by GTC and VitE dietary inclusion [24]. Regarding the color, lightness was increased by the dietary inclusion of GTC (on average by 4.7%) but only compared with GM105 and VitE groups and after 2 days of aerobic packaging [24] (Table 1).

It should be highlighted here that great attention is devoted from consumers to the fatty acid composition of meat for its implications in human health. In fact, a reduction in SFA and an increase in PUFA consumption is of great interest; in particular, the n-3 series exerts beneficial effects in atherosclerosis and other diseases [25]. An increase of n-3 PUFA content in pork meat can be achieved by the dietary supplementation with marine lipid sources such as fish oil, which is rich in eicosapentaenoic acid (EPA) and docosahexaenoic acid (DHA), or plant lipid sources such as linseed meal/oil, which are rich in α-linolenic acid [26,27]. Olive leaves (OL, 0.5% and 1% of the diet) when added in linseed oil-enriched diets affected the fatty acid profile of LD muscle in pigs, only compared with a sunflower oil-rich ration [28]. The OL supplementation in a linseed oil-enriched diet, compared with a sunflower oil-enriched diet, increased the proportion of SFA (on average by 6.8%), but that increase was not observed when compared with a linseed oil VitE-enriched ration. On the other hand, the proportions of MUFA were decreased in the LD muscle of OL compared with sunflower oil-fed pigs (from 3.2% to 2.4%). A decrease n-6:n-3 ratio (by an average value of 84.5%) was observed in the OL compared with sunflower oil-fed animals. Moreover, effects were observed on the fatty acid composition between OL and VitE groups. Due to the high content of linseed oil in n-3 fatty acid, the authors also investigated its impact on meat lipid peroxidation throughout 9 days of storage. Feeding OL led to a decrease in MDA content in LD muscle, both in comparison with sunflower and linseed oil-fed animals, whereas VitE supplementation was more effective than OL in delaying lipid oxidation. Conversely, protein oxidation was affected neither in raw nor cooked meat of pigs fed with linseed oil. Positive effects were also found in OL and VitE groups regarding sensory meat qualities [28] (Table 1).

The dietary supplementation with antioxidant extract (synthetic VitE; natural VitE; flavonoid extract-enriched diet (Flav); and phenolic compound-enriched diet (Phen)) [23] did not affect the fatty acid composition but had an impact on lipid oxidation of pigs LD. More specifically, the TBARS values in Phen- and Flav-fed animals were higher than in the other groups and only similar to those consuming the basal diet [29] (Table 1).

The chemical composition (moisture, protein, lipids, ash, and cholesterol) of rabbit’s LD muscle did not differ when the standard diet (50 mg/kg of VitE) was supplemented with VitE (150 mg/kg), oregano extract (OE, 0.2% of the diet), RE (0.2% of the diet), or oregano and rosemary extract simultaneously (0.1% OE + 0.1% RE of the diet, ORE) [30]. Lower moisture (by 2.0%) and higher protein (by 5.8%) levels were observed in meat from animals consuming VitE compared with OE enriched diets, whereas ORE had only a higher moisture content than VitE fed animals (by 2.0%). Ash content was lower after OE and ORE compared with VitE supplementation (by 8.1%), whereas the rosemary addition lead to intermediate values. However, dietary supplementation with VitE, OE, and RE does not affect the chemical and mineral composition of rabbits’ hind leg meat. Only TBARs values of rabbits’ LD muscle were reduced by dietary supplements. The oxidative stability of raw meat was better preserved (lower TBARS) when VitE or OE, compared with rosemary extract alone or combined with oregano, were added in rabbits’ rations. Dietary supplementation also affected rabbits’ bone traits of the hind leg. Bones of the OE compared with VitE fed animals were heavier, probably because of a heavier femur weight [30] (Table 1). The other groups presented intermediate values for bone weight. The resistance of the femur to fracture and the percentage of meat on the hind leg were the same in all groups [24]. The OEO (0.1% or 0.2% of the diet) compared with VitE (200 mg/kg) had similar antioxidant action in both raw and thermally treated muscle of rabbits [31]. In fact, the dietary supplementation with OEO lowered the MDA values compared with the basal (by 53%), but not to VitE (greater by 44.02%), enriched diet in the LD of rabbits [31] (Table 1). Those results confirm that in rabbits’ tissues, antioxidant activity was improved by the OEO dietary addition.

**Table 1:** Effects of plant feed additives on monogastric meat quality traits.

Trait Evaluated	Species	PFA	Vs. Negative Control	Vs. Positive Control	Reference
Carcass weight	Rabbits	Oregano and Rosemary extracts	↑	↑	[30]
	Swine	Oregano oil, quercetin	↑	↑	[22]
Carcass yield	Rabbits	Oregano and Rosemary extracts	↑	↑	[30]
	Swine	Green tea catechins and grass meal	↑	NS	[24]
	Swine	Oregano oil, quercetin	↑	↑	[22]
Backfat, Fat depth (Shoulder), Ash	Swine	Green tea catechins and grass meal	↑	NS	[24]
Moisture	Rabbits	Oregano and Rosemary extracts	NS	↑	[30]
Protein, Ash	Rabbits	Oregano and Rosemary extracts	NS	↓	[30]
Total fatty acid content	Swine	Rosemary extract	↑	↑	[23]
SFA, n-3 PUFA	Swine	Olive leaves	↑	NS	[28]
MUFA, n-6 PUFA, n-6/n-3, PUFA/SFA	Swine	Olive leaves	↓	NS	[28]
n-6/n-3	Swine	Green tea catechins and grass meal	↓	NS	[24]
pH	Swine	Green tea catechins and grass meal	↓	NS	[24]
	Swine	Oregano oil, quercetin	↑	NS	[22]
a*, b*, L* in MAP	Swine	Green tea catechins and grass meal	↑	NS	[24]
Bone weight	Rabbits	Oregano and Rosemary extracts	NS	↑	[30]
Femur weight	Rabbits	Oregano and Rosemary extracts	NS	↑	[30]
TBARs, MDA	Rabbits	Oregano and Rosemary extracts, Oregano essential oil	↓	↑	[30,31]
	Swine	Rosemary extract, Flavonoid extract-enriched diet, and phenolic compound-enriched extract	↑, NS	↑	[23,29]
	Swine	Green tea catechins	↓	NS,↑	[24,28]
α-tocopherol	Swine	Green tea catechins and grass meal	NS	↓	[24]
	Swine	Olive leaves	↑	↓	
ROS, TBARs	Swine	Oregano oil, quercetin	↓	NS	[22]
SOD, GPx	Swine		NS		
Drip loss	Swine	Oregano essential oil	↓	↓	[21]

Abbreviations: color components (L*, lightness; a*, green-red; b *, blue-yellow); GPx, glutathione peroxidase; MAP, modified atmosphere packaging; MDA, malondialdehyde; MUFA, monounsaturated fatty acids; NS, not significant; PUFA, polyunsaturated fatty acids; ROS, reactive oxygen species; TBARS, thiobarbituric acid reactive substances; SFA, saturated fatty acids; SOD, total superoxide-dismutase.

### 3.2. Ruminants

Detailed information for the studies presented in this section is reported in Table S2 in Supplementary Materials.

#### 3.2.1. Meat Antioxidant Status

Luciano and colleagues [32] partially replaced the barley grain of lambs’ diets with dried citrus pulp as a source of natural VitE, in order to test its effect on their antioxidant capacity. Although these researchers did not use any extra quantity of synthetic VitE (α-tocopherol acetate or α-tocopherol) in the experimental diets, an increase in the concentrations of VitE in liver, plasma, and muscles of lambs fed with the dried citrus pulp was observed. This was not accompanied by any change in lipid oxidation of the above raw tissues. Nevertheless, lambs’ muscles challenged with further stressors, such as the presence of Fe^3+^/Asc, which induce lipid peroxidation, revealed lower TBARS values, which were negatively correlated with the concentrations of VitE in the above tissues. A significantly higher daily intake of VitE (6.23 vs. 12.22 mg) in weaned lambs fed concentrate was found, when they were fed dried tomato pomace on an ad libitum basis [7]. Although the dried tomato pomace did not affect the lambs’ performance, it significantly reduced their concentrate intake. Additionally, the dietary inclusion of dried tomato pomace increased the concentrations of linoleic acid, VitE, and vitamin A in lambs’ meat. Although VitE intake was higher in the dried tomato pomace-fed lambs, no increase in the concentration of this vitamin in their muscles was found. This might be the reason for the unaffected lipid oxidation (as determined by TBARS assay) observed in lambs’ raw meat.

Neither the addition of grape pomace (GP; 51.7 or 103 g/kg DM) nor extra VitE (450 mg/kg DM) in the ewes’ basal diet (50 mg VitE/kg DM) affected the animal performance, carcass characteristics, and meat quality of their suckling lambs [33] (Table 2). However, the proportions of MUFA in the intramuscular fat of suckling lambs were declined significantly, when the goats’ diet, which contained 50 mg VitE/kg DM in the vitamin premix, was supplemented with either GP (51.7 or 103 g/kg DM) or extra VitE (450 mg/kg DM) [27]. In fattening lambs, the dietary supplementation with either VitE (6 g/kg DM) or naringin (1.5 or 3 g/kg DM) resulted in a significant reduction in the TBARS values in their blood plasma [34]. Moreover, both VitE (6 g/kg DM) and (1.5 or 3 g/kg DM) in fattening lambs achieved to restrict the serum concentrations of triglycerides, which occurred due to the fish oil incorporation in their diets [28].

The incorporation of extra VitE (300 mg/kg feed) in lambs diet, further to that already contained in the vitamin premix, improved the oxidative stability and linking sensory scores in their LD muscle compared with those fed either with the basal diet alone or combined with wine extracts (900 mg/kg feed; Table 2) [35]. A significant decline in the lipo-oxidation compounds such as heptanone and 1-octen-3-ol in lambs’ LD was observed when extra VitE (300 mg/kg) was added to the diet [36]. However, this effect was not observed when red wine extract (900 mg/kg) was added [36].

The oxidative stability of *Longissimus thoracis* and *semitendinosus* muscles improved significantly when the cattle were fed simultaneously with VitE (2.8 g/animal/day) and plant extracts (126 g/animal/day), compared with those that consumed VitE (2.8 g/animal/day; Table 2) only [37]. Although lamb meat oxidation was prevented when adding extra VitE (600 mg/kg DM) instead of rosemary diterpenes, the latter had better antimicrobial effects [38,39] (Table 2). In another similar study, the same researchers found that the addition of extra VitE, compared with that of rosemary, has better antioxidant effects on lambs’ meat storage [32]. Only the supplementation with extra VitE (450 mg/kg) has shown the potential to reduce discoloration and lipid oxidation and improved the shelf life in the *Longissimus thoracis* et *lumborum* muscles of lambs due to a reduction in the microbial count on it during storage compare with supplementation with grape seed extract (50 mg/kg), or grape pomace (5%; Table 2) [40].

A subcutaneous injection with saffron petal extract (25 mg/kg body weight (BW)) or VitE (500 mg/kg BW) induced a significant reduction in the cholesterol level in the lambs’ plasma compared with those fed with the basal diet alone or supplemented orally with saffron petal extract (500 mg/kg BW) [41]. Moreover, it should be pointed out here that none of the above treatments affected the antioxidant status of the liver and LD of lambs. In goats, diet supplementation with *Andrographis paniculata* and turmeric acid (0.5%) improved the color parameters of *infraspinatus* muscle and carcass traits [42] (Table 2).

#### 3.2.2. Milk and Fatty Acids Profile

The VitE (7.238 IU) supplementation in the extruded fed cows had no effect on milk yield, milk fat, or protein percentage, but resulted in a moderate effect on milk fatty acids profile [43]. On the other hand, a slight increase in the percentages of C6:0, C18:0, C20:0, and MUFA, and a decrease in C16:0, C22:5n-3, and SFA in the milk fat of extruded fed cows were found when supplemented with plants extracts (191 g) rich in polyphenols, compared with VitE [43]. Thus, the simultaneous supplementation of extruded linseed-fed cows with plant extracts and VitE tented to reduce the impact of VitE alone on the milk’s fatty acids profile, although it had no effect on dairy performance [42]. The milk’s oxidative reducing power increased while both the protein and solids percentages reduced significantly in Yerba mate-fed cows (30 g/kg DM) compared with those fed with (375IU VitE/Kg DM) or without VitE [44] (Table 2).

**Table 2:** Effects on quantitative and qualitative traits of lamb meat and cattle and sheep milk.

Trait Evaluated	Species	PFA	Animal Product	Type of Parameter	Vs. Negative Control	Vs. Positive Control	Reference
Milk concentration of protein and total solids	Bovine	Yerba Mate (Ilex paraguariensis)	Milk	Milk quality	↓	NS	[44]
Total meat (kg)	Goat	Turmeric acid	Meat	Carcass traits	↑	NS	[42]
Breast and flank					NS	↑	
Breast and flank					↑	NS	
Leg, Breast, and flank					↓	↓	
Eye muscle area, Eye muscle depth					↑	NS	
Total meat, breast and flank, Eye muscle area, and depth	Goat	Andrographis paniculata	Meat		↑	NS	[42]
Neck					↑	↑	
Loin					NS	↑	
Breast and flank					↓	NS	
Eye muscle back fat					↓	↓	
pH	Ovine	Rosemary extract (diterpenes)	Meat	cheesy odor	↑	NS	[39]
	Ovine	Rosemary extract (diterpenes)	Meat	cheesy odor	↓	NS	[39]
MDA	Bovine	Plant extract rich in polyphenols	Milk	Ageing: 7 days of storage (modified atmosphere)	↓	NS	[37]
	Goat	Andrographis paniculata	Meat	Lipid oxidation of Infraspinosus muscle	↓	↑	[42]
TBARS	Ovine	Rosemary extract	Meat	Lipid oxidation	NS	↑	[39]
	Ovine	Rosemary extract	Meat		↓	↑	[38]
Pox (d 7)	Ovine	Rosemary extract	Meat		↓	↑	[38]
Antioxidant content	Ovine	Red wine extract	Meat	α-Tocopherol concentration, total phenol content	NS	↓	[35]
Antioxidant content	Goat	Andrographis paniculata	Meat	Vitamin E	↑	↑	[42]
	Bovine	Yerba Mate (Ilex paraguariensis)	Milk	Milk reducing power	↑	NS	[44]
a*	Ovine, Goat	Grape pomace, Turmeric acid	Milk, Meat	Milk, Subcutaneous fat, and meat color	↑	↑	[33,42]
	Ovine	Grape pomace, grape seed extract	Meat	Meat color	NS	↓	[40]
b*	Ovine	Grape pomace	Milk	Subcutaneous fat color	↓	NS	[33]
	Goat	Turmeric acid	Meat	Meat color	↑	↑	[42]
C*	Ovine, Goat	Grape pomace, Turmeric acid	Milk, Meat	Milk, Subcutaneous fat, and meat color	↑	↑	[33,42]
	Ovine	Rosemary diterpenes (carnosic acid and carnosol	Meat		↑	↓	[38]
H*	Ovine	Grape pomace, Rosemary diterpenes (carnosic acid and carnosol), Rosemary extract (diterpenes)	Milk, Meat	Subcutaneous meat and fat color	↓s	NS	[33,38]
	Ovine	Grape pomace, grape seed extract	Meat	Meat color	NS	↓	[42]
L*	Ovine	Grape pomace	Milk	Subcutaneous fat color	↓	↓	[33]
	Goat	Turmeric acid	Meat	Meat color	↑	↑	[42]
	Ovine	Grape pomace, grape seed extract	Meat	Meat color	NS	↑	[42]
	Ovine	Rosemary diterpenes (carnosic acid and carnosol	Meat		↓	↑	[38]
H*	Goat	Andrographis paniculata	Meat	Total meat (kg)	↑	NS	[42]
Color	Ovine	Rosemary extract (diterpenes), Rosemary diterpenes (carnosic acid and carnosol)	Meat	Lean color, fat color	↑	NS	[38,39]
Odor	Ovine	Rosemary extract (diterpenes)	Meat	Serum odour	↑	NS	[39]
	Ovine	Rosemary extract (diterpenes)		Rancid odor	NS	↑	[39]
	Ovine	Rosemary diterpenes (carnosic acid and carnosol	Meat	Rancid odour	↑	↓	[38]
	Ovine	Rosemary diterpenes (carnosic acid and carnosol), Rosemary extract (diterpenes)	Meat	Acid odour	NS↓	NS↓	[38,39]
	Ovine	Rosemary extract (diterpenes)	Meat	Cheesy odor	↓	↓	[39]
	Ovine	Rosemary diterpenes (carnosic acid and carnosol	Meat	Meaty odor, Freshness	↑	NS↓	[38]
	Ovine	Grape pomace, grape seed extract	Meat	Off odor	NS	↓	[42]
Aromatic compounds	Ovine	Red wine extract	Meat	Volatile compounds in omega-3 enriched lamb longissimus dorsi (LD)	NS	↑	[36]
SFA	Ovine	Grape pomace	Milk	Fatty acid profile	NS	↓	[45]
MUFA	Ovine	Grape pomace	Milk	Fatty acid profile	↓	NS	[33]
	Ovine	Grape pomace	Milk	Fatty acid profile	NS	↑	[45]
PUFA	Ovine	Grape pomace	Milk	Fatty acid profile	↑	NS	[33]
n-6/n-3	Ovine	Grape pomace	Milk	Fatty acid profile	↑	↑	[45]
Bacterial count	Ovine	Rosemary extract RE (diterpenes)	Meat	Average bacterial counts of lamb patties packed in different atmospheres Microbial counts of lamb loin kept in retailing conditions	↓	↓	[38,39]
Cooking losses	Ovine	Grape pomace	Milk	Milk yield, composition	↓	↑	[33]

Abbreviations: color components (L*, lightness; a*, green-red; b *, blue-yellow; C* brightness; H*, hue); MDA, malondialdehyde; MUFA, monounsaturated fatty acids; NS, not significant; PFA, plant feed additives; PUFA, polyunsaturated fatty acids; SFA, saturated fatty acids; TBARs, thiobarbituric acid reactive substances.

The addition of either GP (5 or 10 g/100 g diet DM) or VitE (500 mg/100 g diet DM) in a total mixed ration diet, which had a vitamin premix, did not cause remarkable changes in the milk fatty acids profile of ewes. However, the proportions of SFA increased while those of MUFA decreased significantly in the milk of ewes fed with the extra VitE compared with those consuming the other dietary treatments [45].

## 4. Final and General Remarks

The aim of the present review was to summarize the results of the available studies concerning the impact of the use of PFA on livestock productivity and product quality. The main focus was on the antioxidant capacity of PFA as potential alternatives to synthetic vitamins, and in particular, on their effect on livestock animals’ product characteristics.

The literature demonstrated that oregano and rosemary extracts can improve carcass weight and yield more strongly than VitE. In pigs, oregano essential oil was able to reduce drip loss, oxidation products, *Longissimus dorsi* proteins, and ash content in comparison to VitE. The use of rosemary extract, in comparison with VitE, increased total fatty acids content and TBARS values in pigs’ meat. Green tea catechins, compared with VitE, did not affect pigs’ slaughter traits and meat preservation characteristics; the latter also not being affected by flavonoid extract and phenolic compounds. Olive leaves were able to reduce the n-6:n-3 and PUFA:SFA ratio and increased the α-tocopherol levels to a lower extent than the supplementation with VitE in the *Longissimus dorsi*, reducing at the same time lipid peroxidation with positive effects on sensory analysis when compared with basal diet. Hot carcass weight and dressing out were increased in swine by the addition of oregano essential oil and quercetin in a higher proportion than VitE, while the same PFA increased carcass pH with positive effects on lipid oxidation that overcame those of VitE. The administration of plant extract rich in polyphenols decreased the proportion of SFA in bovine milk by increasing, at the same time, those of MUFA, with no specific effects on the oxidative stability but with a similar impact than VitE. Hesperidin and naringin reduced milk MDA content after 14 days of preservation. In ovine meat, red wine extract, compared with VitE, increased volatile compound content in n-3 enriched lamb Longissimus dorsi muscle. Grape pomace showed inconsistent effects on meat color, reducing cooking loss at the same time, decreasing MUFA, and increasing PUFA content. Rosemary extract (diterpenes) is as effective as VitE in reducing the total viable count of *Enterobacteriaceae* and Lactic acid bacteria (LAB) in a short period under different preservation conditions with some positive effects also on color and odor. Moreover, grape pomace in ovine meat depresses the proportions of SFA (with the exception of the C16:0), and MUFA, with some increasing effects on C18:1 and C18:2, generally overcoming the action of VitE on these fatty acids. It also increased the n-6:n-3 ratio in meat lipids. Grape pomace and grape seed extract were not effective in inhibiting bacterial development on ovine meat, while a more sensible effect was exerted by rosemary diterpenes (carnosic acid and carnosol), especially on total viable count and LAB. The latter PFA were also able to positively affect meat color and odor, reducing meat oxidation and rancid odor. In goats, *Andrographis paniculata* and turmeric acid reduced meat MDA content and were very effective in improving color, also in comparison with VitE, with positive effects on carcass traits.

From the available literature on the topic, the majority of the tested PFA can exert some effect mainly in terms of product quality. The main traits affected by the treatments were (i) the antioxidant status of meat which is generally slightly impaired, (ii) the proportion MUFA and PUFA are often increased, with a reduction in meat drip loss, and improved color and odor, and (iii) the n-6:n-3 ratio varied with the product tested and the animal species. Some positive effects were also demonstrated on product microbiologic parameters, particularly in meat. The effects were, in general, not strictly related to the dose and the nature of the product tested and were consistent between the different animals’ species. The activity of PFA can be similar to those of the synthetic antioxidant VitE, even if the mode of action is actually different. The literature comparing natural with synthetic antioxidants for their effects on the animals and products’ oxidative status is still limited, and the experimental protocols and design are very variable, making it difficult to draw specific conclusions on particular natural product categories.

## 5. Conclusions

The dietary PFA supplementation in pigs, rabbits, and ruminants’ diets can exert positive effects on the quality of the derived food products, with variations in their fatty acid content and oxidative status that generally ameliorate the related traits (e.g., odor, color, etc.). Some positive impacts are also exerted on animals’ product yields. The effects of PFA are variable but often similar to those of the synthetic antioxidant VitE, indicating their potential at least as a partial substitute of this vitamin in the diet.

## Data Availability

The data presented in this study are available free of charge for any user on request from the corresponding authors.

## Supplementary Materials

The following are available online at https://www.mdpi.com/article/10.3390/antiox10050780/s1, Table S1 Effects of plant feed additives on monogastric meat quality traits, Table S2 Effects on quantitative and qualitative traits of lamb meat and cattle and sheep milk.
