# Supplementary material for: Plant Feed Additives as Natural Alternatives to the Use of Synthetic Antioxidant Vitamins in Livestock Animal Products Yield, Quality, and Oxidative Status: A Review
Source: Antioxidants (Basel). 2021 May 14;10(5):780. doi: 10.3390/antiox10050780 (PMC8155892; doi:10.3390/antiox10050780)
Supplement: Supplementary file 1 [file antioxidants-10-00780-s001.zip › antioxidants-1174850-supplementary.pdf]

Table S1. Effects of plant feed additives on monogastrics meat quality traits

PFA	Dose extract (% of the diet)	Species	Vitamin confronted	Type of parameter	Trait evaluated	Comparison to negative control: effect (plant product dose)	Comparison to positive control: effect (dose)	Period of study	Reference
**Oregano essential oil**	0.025	Swine	E<br>Transport stressed (TS) Not transported stressed (NTS)	Meat traits	pH 45 min post-mortem	NTS:↓3.17%; TS: ↑4.04%	NS	28 days	Zou et al., 2017
					pHu (24 h), MC (45 min), MC (24 h), EC (45 min), EC (24 h), IMF	NS	NS		
					Drip loss	TS: ↓49.43%	↓46.04%		
**Oregano and Rosemary extracts**	0.2 (OP),0.2 (RP), 0.1OP+ 0.1RP(OPRP)	Rabbits	E	Slaughter traits	CW	↑6.5% (OE) ↑5.98% (OPRP)	↑6.26% (OE) ↑5.74% (OPRP)	50 days	Cardinali et al., 2015
					CY	↑3.4% (O) ↑3.91% (R) ↑1.87% (OR)	↑4.11% (O) ↑4.62% (R) ↑2.57% (OR)		
				Meat composition in LD muscle	Moisture	NS	↑2.19% (O) ↑2.05% (ORRP)		
					Protein	NS	↓6% (O) ↓5.6% (ORRP)		
					Ash	NS	↓8.1% (O) ↓8.82% (ORRP)		
					TBARS	↓25% (O) ↓12.5% ↓16.7% (OPRP)	↑23.53% (O) 17.65% (ORRP)		
					Lipids, CHOL	NS	NS		

				Meat composition in HL meat	Water, protein, lipids, ash, CHOL, Fe, Na	NS	NS		
				Bone traits	Bone weight	NS	↑13.46% (O)		
					Femur weight	NS	↑19.15% (O) ↑14.89% (OPRP)		
					HL weight, Bone % of HL, Meat, Femur fract % of HL, Meat/bones ratio, Femur weight, Femur % of HL, Femur minor diameter, Femur length, Femur fracture	NS	NS		
**Oregano essential oil (OO)**	0.1, 0.2%	Rabbits	E, 200 mg/kg	Lipid oxidation of muscle LD (refrigerated storage, 4 °C)	MDA	↓56.27% (0.1%) ↓49.91% (0.2%)	↑39.13% (O) ↑48.91% (O)	42 days	Botsoglou et al., 2004
**Rosemary extract (RE)**	0.04 (RE), or + 0.02 gallic acid (REG) or + 60 mg/kg of VitE (REE)	Swine	E, 60 mg/kg	Slaughter traits	Slaughter weight, lean meat percentage, post-mortem pH	NS	NS	115, 122 days	Haak et al. (2008)
				Muscle LT and subcutaneous fat composition	Total fatty acid content	↑58.13% (R)	↑48.33% (R)		
					SFA, MUFA, n-6, n-3, PUFA	NS	NS		
				Lipid oxidation of LT muscle	TBARS (d 2) raw meat	NS	NS		

					TBARS (d 5) raw meat	↑57.14% (REG)	↑96.15% (R) ↑153.85% (REG)	
					TBARS (d 8) raw meat	↑67.61% (REG)	↑129.27% (R) ↑190.24% (REG)	
					TBARS (d 2) cooked meat	NS	↑34.82% (REG)	
					TBARS (d 5) cooked meat	NS	↑14.55% (REG)	
					TBARS (d 8) cooked meat	NS	NS	
					Carbonyl and thiol groups in raw and cooked meat during storage	NS	NS	
					CL, DTS	NS	NS	
**Green tea catechins**	0.02	Swine	E	Slaughter traits	Kill-out	C: ↓1.03%; GM105:↑1.05%	NS	Mason et al. (2005)
**GM (grassmeal * enriched diet)**					Backfat	GM105: ↑10.38%	NS	
					CL, DTS	NS	NS	
				Carcass traits	pH24	GM50: ↓4.87%	NS	
					Fat depth (Shoulder)	GM80: ↑38.02%	NS	
					Fat depth (Ham2)	GM80: ↑58.3%	NS	
					pH45, Fat depth (LD), Fat depth (Ham1)	NS	NS	
				Meat composition of *Longissimus dorsi* (LD)	IMF, Protein, Moisture	NS	NS	
					Ash	GM50: ↑14.78%	NS	

	α-tocopherol	NS	↓29%
Lipid oxidation in LD stored under aerobic or MAP: 40% CO_2:60% O_2 conditions at 4 °C for 10 days	TBARS (aerobic condition)	NS	NS
	TBARS in MAP (d 2)	GM105: ↓76.92%	NS
	TBARS in MAP (d 4)	GM105: ↓41.18%; GM50: ↓44.4%	NS
	TBARS in MAP (d 0,6,8,10)	NS	NS
FAC	C20:4n 6	GM105: ↓41.63%; GM50:37.1%	NS
	n-6/n-3	C: ↓56.55%	NS
	from C14:0 to C20:3n-6; from to C20:5n-3 to C22.6n-3; SFA, MUFA, PUFA	NS	NS
	L* (d 2) in aerobic conditions	GM105:↑4.69%	↑4.69%
	L* (d 0,4,8,10) in aerobic conditions	NS	NS
	L* (d 2) in MAP	GM105: ↑7.71%; GM50:↑7.1%; GM80: ↑6.86%	NS
	L* (d 4) in MAP	GM50: ↑6.15%	NS

					L* (d 0,8,10) in MAP	NS	NS	
					a* in aerobic conditions (d 0,2,4,8,10)	NS	NS	
					a* in MAP (d 0,4,8,10)	NS	NS	
					a* in MAP (2)	GM105:↑31.31%	NS	
					b*in aerobic conditions (d 0,2,4,8,10)	NS	NS	
					b*in MAP (2)	GM105: ↑23.74%; GM50: ↑16.63%; GM80: ↑23.9%	↑17.8%	
					b*in MAP (4)	GM105: ↑22.59%	NS	
					b* in MAP (d 0,8,10)	NS	NS	
**Flavonoid extract-enriched diet (AFlav); and phenolic compound-enriched extract (APhen)**	0.2	Swine	E, 200 mg/kg	FAC	C20:1 n-9	NS	↓18.48% (Aflav)	Gonzalez and Tejeda (2007)
					Other FA	NS	NS	
				Lipid oxidation	MDA LD muscle (refrigerated storage, 10 d)	NS	↑ (Aflav, Aphen)	
**Olive leaves**	0.5, 1	Swine	E in diet enriched with linseed oil	FAC of LD muscle	From C12:0 to C16:1	NS	NS	Botsoglou et al. (2012)

C18:0	↑15.27% (0.5%) ↑17.24% (1%)	NS
cis-9 C18:1	NS	NS
C18:2n−6	↓26.74% (0.5%) ↓28.83% (1%)	NS
C18:3n−3	↑413% (0.5%) ↑446.4% (1%)	NS
C20:0, C20:1	NS	NS
C20:3n−6	↓41.18% (0.5%) ↓23.53% (1%)	NS
C20:4n−6	↓52.1% (0.5%) ↓41.7% (1%)	NS
C20:5n−3	↑460% (0.5%) ↑580% (1%)	NS
C22:5n−3	↑216.7% (0.5%) ↑166.7% (1%)	NS
C22:6n−3	NS	NS
SFA	↑5.86% (0.5%) ↑7.76% (1%)	NS
MUFA	↓3.22% (0.5%) ↓2.37% (1%)	NS
PUFA	NS	NS
n−6 PUFA	↓28.22% (0.5%) ↓29.15% (1%)	NS

					n−3 PUFA	↑350.27% (0.5%) ↑373.22% (1%)	NS		
					n− 6:n− 3	↓84.05% (0.5%) ↓85.01% (1%)	NS		
					PUFA:SFA	↓7.69% (0.5%) ↓8.97% (1%)	NS		
					α-Tocopherol	LD: ↑6.41% (0.5%)	↓70.57% (0.5%) ↓69.15% (1%)		
				Lipid oxidation	MDA during 9 days of storage in LD muscle	LD: ↓0.5%>1%; S: ↓0.5%>1% *P*<0.05	↑0.5%>0.1%		
				Protein oxidation	Carbonyl groups	NS	NS		
				Sensory analysis		Positive effects	Positive effects without NS		
**OO, quercetin (QU)**	0.025	Swine	E	Carcass traits	Pre-slaughter BW, Carcass straight length, Carcass slanting length, Back-fat thickness, EC (45 min), EC (24 h), Opto-Star	NS	NS	28 days	Zou et al. (2016)
					Hot CWs (after 5 hours of transportation)	↑8.25% (OO)	↑7.14% (OO)		
					Dressing out	↑8.16% (OO)	↑6.89% (OO)		

	pHi (45 min)	↑5.56% (OO) ↑4.9% (QU)	NS
	pHu (24 h)	↑3.18% (Q)	NS
AA status muscle	ROS	↓20.96% (QU) ↓17.88% (OO)	NS
	TBARs	↓20.39% (QU) ↓19.42% (OO)	NS
	T-SOD,GPx	NS	NS

Abbreviations: AA, antioxidant activity; BW, body weight; CHOL, cholesterol; CL, carcass lean; CY, carcass yield; CW, carcass weight; DTS, days to slaughter; EC, electrical conductivity; FAC, fatty acid composition; GPx, glutathione peroxidase; HL, hind leg; IMF, intramuscular fat; LD, *Longissimus dorsi* muscle; LT, *Longissimus thoracis* muscle; MAP, modified atmosphere packaging; MC, meat colour; MDA, malondialdehyde; NS, not significant; NTS, not transport stressed group; pHi, pH immediately; pHu, pH ultimately; ROS, reactive oxygen species; TBARS, thiobarbituric acid reactive substances; T-SOD, total superoxide-dismutase.

Table S2 Effects on quantitative and qualitative traits of lamb meat and cattle and sheep milk

PFA	Dose extract	Species	Vitamin confronted	Animal product	Type of parameter	Trait evaluated	Comparison to negative control: effect (plant product dose)	Comparison to positive control: effect (dose)	Period of study (d, days; wk, weeks)	Reference
**Plant extract rich in polyphenols**	1 along with 375 UI of Vitamin E (PERP)	Bovine	E, 375 IU/kg	Milk	FAC of milk	Total SFA	2 control diets: 1 with maize silage (C), 1 with Linseed oil (CL); C: ↓25.99% CL: ↓3.31%	↓6.52%	5 weeks	Ferlay et al. (2010)
						Total MUFA	C:↑78.28% CL: ↑3.94%	↑9.67%		
**Plant extract rich in polyphenols**	12.6 along with 2800 mg/kg	Bovine	E, 2800 mg/kg	Meat	Oxidative stability of semitendinosus (ST) steaks					Gobert et al. (2010)
					Ageing in carcass	MDA ST muscle after 14 d (V) in a tray under-vacuum packaging	NS	NS		
						4 d aerobic packaging (A)	NS	NS		
						7 d under modified atmosphere packaging (70:30, O_2/CO_2) (ageing in carcass) (MMA)	↓72.6 3%	NS		
					Ageing under-vacuum	V	NS	NS		
						A	NS	NS		

						MA	↓80.74%	NS		
					Oxidative stability of LT muscle		NS	NS		
					Ageing in carcass		NS	NS		
							↓67.07%	NS		
					Ageing under-vacuum	V	NS	NS		
						A	NS	NS		
						MA	↓84.55%	NS		
						TAS	NS	NS		
**Saffron petal extract**	0.0025,0.05	Ovine	E, 225 IU/kg	Meat	Carcass traits	Hot CW, Cold CW	NS	NS	56	Alipour et al. (2019)
**Red wine extract**	0.09	Ovine	E	Meat	Volatile compounds in omega-3 enriched lamb *longissimus dorsi* (LD)	2-Heptanone	NS	↑30.61%		Rivas-Canedo et al. (2013)
						3,5-Octadien-2-one	NS	↑81.16%		
						2-Penten-1-ol	NS	↑90.24%		
						2-Octen-1-ol	NS	↑47.44%		
						1-Penten-3-ol	NS	↑58.5%		
						1-Octen-3-o	NS	↑60.81%		
						Pentane	NS	NS		
						Heptane	NS	NS		
						Benzene	NS	↑45.1%		
						Phenylacetaldehyde	NS	↑37.1%		
						Ethylbenzaldehyde	NS	↑63.6%		
						Trimethylamine	NS	↑107.82%		
						Unknown compound LRI 1093	NS	↑115.1%		

						2-Propanone	NS	↓36.25%	
						Dimethylsulfide	NS	↓31.74%	
						2,3-Butanedione	↑86.37%	NS	
						2,3-Butanediol, 1-Hexanol	NS	NS	
**Grape pomace**	5, 10	Ovine	E, 500 mg/kg	Milk	Milk yield, composition		NS	NS	Gómez-Cortés et al. (2018)
				Meat	Carcass traits	Slaughter age (days)	NS	NS	
						Cold carcass weight, Carcass yield	NS	NS	
					pH, color, cooking losses, texture and chemical composition of suckling lamb meat.	Meat pH 24 h post slaughter *L. thoracis*	NS	NS	
					Rectus abdominis colour	L*, b*, H*	NS	NS	
						a*	↑ 32.62% (5%)	↑51.47% (5%)	
						C*	↑12.81% (5%)	↑20% (5%)	
					L. thoracis et *lumborum* colour	L*, a*, C*	NS	NS	
						b	↓28.81% (5%) ↓23.88% (10%)	NS	
						H*	↓26.81% (5%) ↓25.36% (10%)		
					Subcutaneous fat colour	L*	↓2.29% (10%)	↓2.68% (10%)	
						a*,b*, H*, C	NS	NS	

	Warner-Bratzler shear force	NS	NS
	Cooking losses (%) (WHC	↓11.2% (5%)<br>↓20.75% (10%)	↑13.23% (5%)
	Moisture, fat, protein	NS	NS
FA composition of meat	iso C14:0	↓26.32% (5%)<br>↓57.89% (10%)	↓50% (10%)
	SFA	NS	NS
MUFA	*trans*-3 C16:1	↑50% (5%)	↑100% (5%)
	cis-9 C17:1	NS	↑14.81% (5%)
	cis (9+10)+*trans*-15 C18:1	↓10.83% (5%)<br>↓9.75% (10%)	NS
	cis-11 C18:1	↓11.22% (5%)<br>↓14.15% (10%)	NS
	cis-14 C18:1	↑66.7% (5%)	NS
	cis-15 C24:1	↓40.82% (5%)<br>↓53.06% (10%)	↓41.03% (10%)
PUFA	*trans*-9 *cis*-11 C18:2	↑100% (5%)	↑100% (5%)
	cis-9 *trans*-11 *cis*-15 C18:3	↑20.62% (5%)	NS
	C20:2 n-6	↑28.57% (5%)	NS
	MUFA	↓7.18% (5%)<br>↓5.85% (10%)	NS
	PUFA	NS	NS
	trans-C18:1	NS	NS

**Red wine extract**	0.09	Ovine	E, 300 mg/kg	Meat	α-Tocopherol concentration, total phenol content and pH values of n-3 enriched LD muscle	α-Tocopherol	NS	↓58.26%		Muino et al. (2014)
						Polyphenols	NS	NS		
						pH24	NS	NS		
					FA composition (%) of n-3 enriched LD muscle	Mean (12 d) C22:5n-3	↑40.98%	NS		
					Sensory evaluation of n-3 enriched LD muscle	OdourOdour, texture, flavour, overall linking	NS	NS		
Andrographis paniculata **(AP), Turmeric acid (TU)**	0.5	Goat	E	Meat	Lipid oxidation of IS	MDA	↓23.08% (AP)	↑2.36% (AP)	100	Karami et al. (2010)
					Colour	L*	↑10.74% (TU)	↑14.35% (TU)		
						a*	↑46.17% (TU)	↑42.86% (TU)		
						b*	↑76.55% (TU)	↑76.32% (TU)		
						C*	↑60.54% (TU) ↑9.01% (	↑58.79%% (TU) ↑7.82% (		
						Hue	↑13.12% (TU)	↑14.77% (TU)		
						Vitamin E	↑23.33% (TU)	↑25.42% (TU)		
						Drip loss, cooking loss, tenderness	NS	NS		

**Rosemary extract –RE- (diterpenes)**	0.06	Ovine	E, 600 mg/kg	Meat	Average bacterial counts of lamb patties packed in different atmospheres (air, vacuum and high O_2/CO_2) and stored at 4 °C for up to 7 days	TVC air (1 d)	↓9.9%	NS	4 months	Ortuno et al. (2017)
						TVC air (4 d)	↓8.52%	↓8.78%		
						TVC (7d)	NS	NS		
						TVC vacuum (1 d)	NS	NS		
						TVC vacuum (4 d)	↓13.09%	↓8.35%		
						TVC vacuum (7 d)	NS	NS		
						TVC High O_2/CO_2 (1 d)	NS	NS		
						TVC High O_2/CO_2 (4 d)	↓14.1%	↓12.90%		
						TVC High O_2/CO_2 (7 d)	↓6.05%	↓7.22%		
						Pseudomonas spp	NS	NS		
						ENB air (1 d)	↓22.57%	↓17.08%		
						ENB air (4, 7 d)	NS	NS		
						ENB vacuum (1 d)	↓21.58%	↓19.92%		
						ENB vacuum (4, 7d)	NS	NS		
						ENB High O_2/CO_2 (1 d)	↓24.03%	↓19.67%		
						Brochothrix thermosphacta	NS	NS		
						LAB air (1 d)	↓20.19%	↓12.37%		

	LAB air (4 d)	↓18.92%	↓16.43%
	LAB air (7 d)	↓12.25%	↓12.11%
	LAB vacuum (1 d)	↓17.99%	↓14.1%
	LAB vacuum (4 d)	↓27.31%	↓18.1%
	LAB vacuum (7 d)	↓11.82%	↓15.82%
	LAB vacuum High O_2/CO_2 (1 d)	↓16.7%	↓14.32%
	LAB vacuum High O_2/CO_2 (4 d)	↓24.95%	↓22.95%
	LAB vacuum High O_2/CO_2 (7 d)	↓11.03%	↓13.61%
Average values of pH, colour, (TBARs) of lamb patties packed in different atmospheres (air, vacuum and high O_2/CO_2) and	pH air (7 d)	↑3.74%	NS
stored at 4 °C for up to 7 days			
	pH air (1, 4 d)	NS	NS
	pH vacuum (1,4, 7 d)	NS	NS
	pH High O_2/CO_2 (1, 4 d)	NS	NS
	pH High O_2/CO_2 (7 d)	↑3.37%	NS
	Lightness	NS	NS
	Hue air (1 d)	NS	NS
	Hue air (4 d)	NS	↑33.47%

	Heu air (7 d)	↓12.64%	NS
	Heu vacuum (1, 4, 7 d)	NS	NS
	Heu High O_2/CO_2 (1, 4 d)	NS	NS
	Heu High O_2/CO_2 (7 d)	↓30.35%	NS
	Chroma	NS	NS
Lipid oxidation	TBARS air (d 1)	NS	↑81.25%
	TBARS air (d 4, 7)	NS	NS
	TBARS vacuum (d 1)	NS	↑87.5%
	TBARS vacuum (d 4)	NS	↑31.58%
	TBARS vacuum (d 7)	NS	↑116.7%
	TBARS High O_2/CO_2 (d 1)	NS	↑92.41%
	TBARS High O_2/CO_2 (d 4)	NS	↑40.37%
	TBARS High O2/CO2 (d 7)	↓24.29%	NS
Average sensory scores of lamb patties packed in different atmospheres (air, vacuum and high O_2/CO_2) and stored at 4 °C for up to 7 days	Lean colour air (1 d)	NS	↓12.2%
	Lean colour air (4 d)	NS	NS
	Lean colour air (7 d)	↑38.1%	NS
	Lean colour vacuum (1 d)	NS	NS

Lean colour vacuum (4 d)	↑16.95%	NS
Lean colour vacuum (7 d)	↑12.5%	NS
Lean colour High O_2/CO_2 (1 d)	NS	NS
Lean colour High O_2/CO_2 (4 d)	↑20.73%	NS
Lean colour High O_2/CO_2 (7 d)	↑84.92%	NS
Serum odour air (1 d)	NS	↓9.3%
Serum odour air (4 d)	NS	NS
Serum odour air (7 d)	NS	NS
Serum odour vacuum (1 d)	NS	NS
Serum odour vacuum (4 d)	↑31.16%	NS
Serum odour vacuum (7 d)	↑22.8%	↑18.53%
Serum odour High O_2/CO_2 (1 d)	NS	NS
Serum odour High O_2/CO_2 (4 d)	↑32.81%	NS
Serum odour High O_2/CO_2 (7 d)	↑30.25%	NS
Rancid odour air (1 d)	NS	↑19.12%
Rancid odour air (4 d)	NS	↑18.52%
Rancid odour air (7 d)	NS	NS
Rancid odour vacuum (1, 4, 7 d)	NS	NS

						Rancid odour High O_2/CO_2 (1,7 d)	NS	NS	
						Rancid odour High O_2/CO_2 (4 d)	↓22.11%	NS	
						Acid odour air (1, 4 d)	NS	NS	
						Acid odour air (7 d)	↓27.47%	NS	
						Acid odour vacuum (1,4, 7d)	NS	NS	
						Acid odour High O_2/CO_2 (1,4 d)	NS	NS	
						Acid odour High O_2/CO_2 (7 d)	↓24.72%	↓22.49%	
						Cheesy odour air (1,4 d)	NS	NS	
						Cheesy odour air (7 d)	↓28.32%	↓28.32%	
						Cheesy odour vacuum (1,4 d)	NS	NS	
						Cheesy odour vacuum (7 d)	↓35.15%	↓29.95%	
						Cheesy odour High O_2/CO_2 (1 d)	NS	NS	
						Cheesy odour High O_2/CO_2 (4 d)	↓22.54%	↓19.71%	
						Cheesy odour High O_2/CO_2 (7 d)	↓29.63%	↓27.85%	
**Grape pomace**	5, 10	Ovine	E, 500 mg/kg	Milk	FAC				Manso et al. (2016)
					SFA	C4:0	NS	↓5.71% (5%)	
						C6:0	NS	↓10.44% (5%)	

	C8:0	↓11.60% (10%)	↓10.56% (5%) ↓17.01% (10%)
	C10:0	↓13.1% (10%)	↓17.87% (10%)
	C12:0	↓12.95% (10%)	↓15.58% (10%)
	C16:0	↑5.79% (5%) ↑6.05% (10%)	NS
	C17:0	↓11.86% (10%)	NS
	C23:0	↓25% (5%) ↓25% (10%)	↓25% (5%) ↓25% (10%)
	C24:0	↓25% (5%)	NS
	17:0 iso	↓12% (10%)	↓8.33% (10%)
MUFA	*cis*-11 C12:1	↓50% (10%)	↓66.7% (10%)
	cis-7 C16:1	↓5.88% (5%)	↑14.29% (5%) ↑21.43% (10%)
	cis-12 C16:1	↓33.3% (5%,10%)	NS
	cis-9 C17:1	NS	↑23.08% (5%) ↑23.08% (10%)
	trans-C15:1	↓11.1% (10%)	↓11.1% (5%,10%)
	trans-13 C16:1	↑33.3% (10%)	↑33.3% (10%)
UFA	*trans*-9,*cis*-12,*cis*-15 C18:3	↑50% (10%)	NS
	C18:3n-3	NS	↓18.64% (10%)
	C20:4n-6	↑15.39% (10%)	↑15.39% (10%)

	SFA	NS	↓2.47% (5%) ↓4.9% (10%)
	MUFA	NS	↑7.12% (5%) ↑11.87% (10%)
	MUFA cis	NS	↑12.38% (10%)
	n-6/n-3	↑23.23% (10%)	↑16.78% (5%) ↑33.57% (10%)
	De novo synthesis fatty acids	NS	↓8.18% (10%)
Cis- and *trans*- C18 MUFA and the conjugated and nonconjugated 18:2 isomers	*Cis*- and *trans*- 18:1		
	cis-9 18:1	NS	↑12.1% (10%)
	cis-12 18:1	↑36.73% (10%)	↑48.9% (10%)
	cis-14 18:1	NS	↑25% (10%)
	cis-15 18:1	NS	↑23.1% (10%)
	trans-8 18:1	↑22.2% (10%)	↑22.2% (10%)
	trans-9 18:1	↑12.5% (10%)	↑12.5% (10%)
	trans-12 18:1	↑14.52% (10%)	↑14.52% (10%)
	trans-13 + trans-14	↓16.88% (5%)	NS
	trans-16 18:1	↓18.92% (5%)	↑9.1% (10%)
Non-conjugated C18:2	*cis*-9,*trans*-13 + *trans*-8,*cis*-12	↓13.04% (5%)	↑22.5% (10%)
	trans-8,*cis*-13	↓20% (5%)	↑25% (10%)

						trans-9,*cis*-12	↑33.3% (10%)	↑33.3% (10%)	
						trans-11,*cis*-15	↑33.75% (5%)<br>↑36.25% (10%)	NS	
						cis-9,*cis*-12	↑10.27% (10%)	↑11.48% (10%)	
						cis-9,*cis*-15	↑33.3% (10%)	↑33.3% (10%)	
					Conjugated C18:2	*trans*-13,*trans*-15 + *trans*-12,*trans*-14	↑20% (10%)	↑50% (10%)	
						Other *trans-trans*	↓25% (5%)	NS	
**Grape pomace, grape seed extract**	0.005 (GSE), 5 (GP)	Ovine	E, 500 mg/kg	Meat	Microbial counts (log cfu g−1) (mean values) on m. *longissimus thoracis et lumborum* lambs during refrigerated storage at 2 °C	TVC (0, 4, 7 d)	NS	NS	Guerra- Rivas et al. (2016)
						TVC (11 d)	NS	↑49.28% (GSE)<br>↑46.89% (GP)	
						TVC (14 d)	↑32.1% (GSE)<br>↑29.77% (GP)	↑81.26% (GSE)<br>↑78.1% (GP)	
						ENB (0.4,7 d)	NS	NS	
						ENB (11 d)	NS	↑89.2% (GSE)	
						ENB (14 d)	NS	↑187.26% (GSE)	
						LAB (0, 4, 7, 11 d)	NS	NS	

	LAB (14 d)	NS	↑319.38% (GSE)<br>↑230.63% (GP)
	Pseudomonas spp (0,4,7 d)	NS	NS
	Pseudomonas spp (11 d)	NS	↑116.85% (GSE)<br>↑113.86% (GP)
	Pseudomonas spp (14 d)	↑33.67% (GSE)<br>↑34.52% (GP)	↑105.19% (GSE)<br>↑106.5% (GP)
	Brochothrix thermosphacta (0,4,7,14 d)	NS	NS
	Brochothrix thermosphacta (11 d)	↑106% (GSE)	NS
Lipid oxidation	MDA	NS	NS
Meat colour	lightness (L*) from 11 d	NS	↑5.81% (GSE)<br>↑4.54% (GP)
	Redness (a* value) (11, 14 d)	NS	↓18.64% (GSE)<br>↓23.87% (GP)
	Yellowness (b*)	NS	NS
	Chroma	NS	NS
	H*	NS	↓(GSE)<br>↓(GP)<br>*P*<0.05
	Appearance scores (7 d)	↓ (GSE)	↓ (GSE)<br>*P*<0.05
	From d 11 onwards	NS	↑(GSE)<br>↑(GP)
	Off-odour from 11 d onwards	NS	↑GSE>GP<br>(P< 0.05)

**Rosemary diterpenes (carnosic acid and carnosol**	0.00005	Ovine	E, 600 mg/kg	Meat	Microbial counts of lamb loin kept in retailing conditions	TVC (d 0)	↓15.31%	↓12.47%	Ortuno et al. (2015)
					for up to 18 days				
						TVC (d 7)	↓14.03%	↓17.34%	
						TVC (11, 14 d)	NS	NS	
						TVC (d 18)	↓11.55%	↓12.55%	
						LAB (d 0)	NS	NS	
						LAB (d 7)	↓28.32%	↓35.94%	
						LAB (d 11)	↓32.95%	↓34.27%	
						LAB (d 14)	↓35.56%	↓33.17%	
						LAB (d 18)	↓34.19%	↓33%	
						ENB (0,7, 11,14, 18 d)	NS	NS	
					Colour	L* (0, 7,11 d)	NS	NS	
						L* (14 d)	↓5.54%	↑4.98%	
						L* (18 d)	NS	↑10.96%	
						C* (0,7 d)	NS	NS	
						C (11 d)	↑31.3%	NS	
						C (14 d)	↑28.57%	↓16.7%	
						C (18 d)	NS	↓30.41%	
						H* (d 0)	NS	NS	
						H* (d 7)	NS	↑25.13%	
						H* (d 11)	↓40.05%	NS	
						H* (d 14)	↓42.86%	NS	
						H* (d 18)	NS	↑165.48%	
					pH		NS	NS	
					a*		NS	NS	
					Lipid oxidation	TBARS (d 0)	NS	NS	
						TBARS (d 7)	↓32.68%	↑597.3%	
						TBARS (d 11)	↓32.96%	↑644.64%	

	TBARS (d 14)	↓32.62%	↑607.04%
	TBARS (d 18)	↓32.39%	↑434.95%
	Pox (protein oxidation) (d 0)	↓18.99%	NS
	Pox (d 7)	↓19.51%	↑41.34%
	Pox (d 11)	↓25.94%	↑45.39%
	Pox (d 14)	↓34.02%	NS
	Pox (d 18)	↓31.61%	↑50.63%
Appearance and odour of lamb loin kept in retailing conditions for up to 18 days	Lean colour (0,7 d)	NS	NS
	Lean colour (11 d)	↑62.99%	NS
	Lean colour (14 d)	↑112.74%	↓24.94%
	Lean colour (18 d)	NS	↓42.5%
	Fat colour (0,7 d)	NS	NS
	Fat colour (11 d)	↑23.74%	NS
	Fat colour (14 d)	↑39.27%	NS
	Fat colour (18 d)	NS	↓20.6%
	Meaty odour (0 d)	NS	NS
	Meaty odour (7 d)	↑16.06%	NS
	Meaty odour (11 d)	↑71.86%	NS
	Meaty odour (14 d)	↑91.82%	↓32.67%
	Meaty odour (18 d)	NS	↓51.06%
	Rancid odour (0 d)	NS	NS

						Rancid odour (7 d)	↓33.33%	NS	
						Rancid odour (11 d)	↓45.73%	NS	
						Rancid odour (14 d)	↓40.26%	↑93.28%	
						Rancid odour (11 d)	↓16.05%	↑100.56%	
						Acid odour (0, 7, 11 d)	NS	NS	
						Acid odour (14 d)	↓16.55%	NS	
						Acid odour (18 d)	NS	↓15.24%	
						Freshness (0 d)	NS	NS	
						Freshness (7 d)	↑10.62%	NS	
						Freshness (11 d)	↑73.33%	NS	
						Freshness (14 d)	↑103.01%	↓29.50%	
						Freshness (18 d)	NS	↓45.58%	
**Yerba Mate (Ilex paraguariensis)**	3, 3 + VitE	Bovine	E, 375 IU	Milk	Milk reducing power		↑	NS	Santos et al. (2019)
						Polyphenols,conjugated dienes, TBARS	NS	NS	
						Milk yield			
						Milk composition (fat, protein, lactose, and total solids), urea	NS	NS	
						Milk concentration of protein and total solids	↓	NS	

**Andrographis paniculata (AP), Turmeric acid (TU)**	0.5	Goat	E	Carcass traits	Hot carcass (kg), cold carcass (%), cold carcass (kg), dressing out		NS	NS	100	Karami et al. (2010)
						Total meat (kg)	↑14.19% (TU)<br>↑13.53% (AP)	NS		
						Total meat (%)	↑6.94% (AP)	NS		
						Breast and flank	NS	↑15.99% (TU)		
					Least square means of proportions (%) of meat, bone, subcutaneous fat and intramuscular fat in different areas of the carcass of goats	Breast and flank	↑6.53% (TU)<br>↑5.87% (AP)	NS		
						Neck	↑17.06% (AP)	↑26.67% (AP)		
						Leg	↓9.78% (TU)	↓9.78% (TU)		
						Loin	NS	↑18.55% (AP)		
						Breast and flank	↓8.72% (TU)<br>↓5.23% (AP)	↓7.1% (TU)		
						Eye muscle area	↑18.63% (TU)<br>↑17.65% (AP)	NS		
						Eye muscle depth	↑21.54% (TU)<br>↑15.39% (AP)	NS		

	Eye muscle back fat	↓23.14% (AP)	↓16.22% (AP)

Abbreviations: CW, carcase weight; ENB, Enterobacteriaceae; FAC, fatty acid composition; IS, *Infraspinatous* muscle; LAB, lactic acid bacteria; LD, *Longissimus dorsi* muscle; LT, *Longissimus thoracis* muscle; MDA, malondialdehyde; MUFA, monounsaturated fatty acids; NS, not significative; PUFA, polyunsaturated fatty acids; SFA, saturated fatty acids; TBARs, thiobarbituric acid reactive substances; TVC, total viable count; UFA, unsaturated fatty acids.
